# Special Issue “Advances in Gastrointestinal and Liver Disease: From Physiological Mechanisms to Clinical Practice”

**DOI:** 10.3390/jcm11102797

**Published:** 2022-05-16

**Authors:** Gian Paolo Caviglia, Davide Giuseppe Ribaldone

**Affiliations:** Division of Gastroenterology, Department of Medical Sciences, University of Torino, 10124 Torino, Italy; gianpaolo.caviglia@unito.it

It is an exciting time for gastroenterology and hepatology. New drugs have entered the market and changed the natural course of several diseases (in particular, hepatitis C and inflammatory bowel disease, IBD), and others are expected in a few years (for example, nonalcoholic fatty liver disease, NAFLD) [1]. Although the identification of the cause of most chronic dysimmune diseases is far from the goal, the daily research that is born in laboratories brings into clinical practice new mechanisms of action and biomarkers useful for personalizing patient management [2,3].

In the next few lines, we want to summarize the most important breakthroughs in the field of gastroenterology and hepatology in the last years.

Hepatis C virus (HCV) treatment is the most important revolution of the past eight years. Before the introduction of direct acting antivirals (DAA), the efficacy and tolerability of interferon-based regimes was far from satisfactory [4]. In 2014, the FDA approved the first all-DAA regimen with sofosbuvir/ledipasvir and sofosbuvir/simeprevir after three clinical trials indicated that DAAs could be administered on their own for HCV genotype 1 treatment with sustained virological response (SVR) rates of 94–99% and significantly fewer adverse effects [5]. The target to eliminate HCV by 2030 no longer appears to be a dream, at least in some countries [6]. Currently under development are a number of new antivirals that target the distinct stages of the HBV life cycle. The goal of these medications, similar to HCV infection, is to cure the infection completely, rather than only inhibiting it. The ultimate goal should be infection control (functional cure) or eradication (complete cure) [7]. Pegylated interferon is the only treatment for chronic hepatitis D (CHD) that has been suggested by professional societies (but not licensed by Drug Regulatory Agencies); it has poor efficacy, and valid CHD treatment has remained an unmet medical need [8]. The goal of current therapeutic attempts is to deprive the HDV of the HBsAg functions that are essential to its life cycle. Three therapy options are currently being tested. Because the HBsAg enters hepatocytes via the sodium taurocholate cotransporting polypeptide (NTCP) on the cell membrane, medications that block the NTCP may prevent the HDV from entering the cells. Because the construction of the HDV virions needs the farnesylation of the large HD antigen by the host, interference with this cellular process could cause the viral assembly to be disrupted. Because the HDV must encapsidate in the HBsAg coat before being released into the bloodstream, nucleic acid polymers (NAPs) that appear to block the formation of subviral HBsAg particles may prevent the HDV from being released into the bloodstream [4].

Regarding IBD, unfortunately a specific cause has not yet been discovered and genetic, environment, immune system, permeability, and microbiome are the main actors described as the cause [9]. Translational research is the key to the basic research of bedside applications. Only in the last 5 years have three new mechanisms of action entered the market: anti-integrin α1β4 with vedolizumab, anti-IL12/23 with ustekinumab, and anti-JAK with tofacitinib. In the next few years several new drugs are expected: anti-IL23, S1P1 regulators, and the more selective anti-JAK [10]. Although we are far from definitively healing these patients, we can try to significantly improve their quality of life, bringing it closer to that of people who do not suffer from these diseases.

In gastrointestinal endoscopy, technology advancement is expressing its maximum potential. The application of artificial intelligence, new techniques, such as submucosal dissection, full thickness resections, and others, are now a reality and we are only at the beginning of the noninvasive treatment of several diseases that once required surgical resection [11].

In this Special Issue, entitled “Advances in Gastrointestinal and Liver disease: From Physiological Mechanisms to Clinical Practice” we welcome frontier papers about novelties in the field of translational research and the clinical management of gastrointestinal and hepatological diseases.

Several papers have already been published in the Special Issue. The etiology of NALD is not yet fully understood and there is a lack of noninvasive biomarkers enabling the differentiation of liver disease severity. Armandi and colleagues conducted a retrospective, cross-sectional investigation to explore the mechanistic involvement of the myokine irisin in a population of biopsy-established NAFLD in the absence of severe metabolic diseases (obesity and type 2 diabetes mellitus) [12]. They discovered that people with severe fibrosis had considerably greater irisin levels. They also discovered a link between circulating irisin and the new collagen remodeling markers PRO-C3 and PRO-C6. The findings point to a synergistic link between irisin and liver fibrogenesis, the hepatic response to inflammatory damage. This supports the idea that irisin is a marker for a more severe phenotype of liver disease, as evidenced by the higher irisin levels found in those with advanced fibrosis. Still, regarding NAFLD, according to research by Gidaro et al., patients with NAFLD have higher levels of C-reactive protein, fibrinogen, PAI-1, von Willebrand factors, and F VII, all of which are linked to an increased risk of thrombosis [13]. Despite having been overtaken by NAFLD in the developed world, HBV infection is a serious health concern [14]. The findings of Caviglia et al. demonstrated that measuring baseline serum HBsAg and the extent to which HBsAg dropped throughout therapy with third-generation nucleot(s)ide analogs can help select chronic HBV patients who are more likely to achieve functional cure [15].

IBD in children is becoming more common around the world and the onset age is getting younger [16], as a consequence, innovative medicinal strategies are essential. The first study on vedolizumab treatment in children with very early onset IBD was published by Fabiszewska et al., and it demonstrated the safety and effectiveness of this anti-integrin agent in the studied group: a clinical response after induction therapy with three doses of vedolizumab was observed in more than 40% of patients [17].

Neuroendocrine tumors (NETs) are diverse malignancies that emerge from systemic endocrine and nerve cells and have a wide range of pathological and clinical features. Their prevalence has been rising [18]. Lymph node metastases can be surgically removed, which can improve prognosis; however, other metastases, which are generally not suggested for surgery, are difficult to remove, underscoring the importance of preoperative diagnosis. In the study of Kohno et al., according to the 2019 WHO classification, factors connected to gastroenteropancreatic-NET metastases were studied. Tumor grade and vascular invasion were revealed to be the most relevant factors. Venous invasion was found to be more strongly associated with metastasis than lymphatic invasion, suggesting that pathological investigation of lymphatic invasion may be difficult.

As the Guest Editors, we are looking forward for other original studies and accomplished reviews regarding recent innovation in gastroenterology and hepatology.
